# Integrating a health-related-quality-of-life module within electronic health records: a comparative case study assessing value added

**DOI:** 10.1186/1472-6963-12-67

**Published:** 2012-03-19

**Authors:** Christopher M Shea, Jacqueline R Halladay, David Reed, Timothy P Daaleman

**Affiliations:** 1 Department of Health Policy and Management, Gillings School of Global Public Health, University of North Carolina at Chapel Hill, Chapel Hill, NC, USA; 2 Cecil G. Sheps Center for Health Services Research, University of North Carolina at Chapel Hill, Chapel Hill, NC, USA; 3 Department of Family Medicine, School of Medicine, University of North Carolina at Chapel Hill, Chapel Hill, NC, USA

**Keywords:** Electronic health records, Quality of life, Clinical informatics, Primary care

## Abstract

**Background:**

Health information technology (HIT) applications that incorporate point-of-care use of health-related quality of life (HRQL) assessments are believed to promote patient-centered interactions between seriously ill patients and physicians. However, it is unclear how willing primary care providers are to use such HRQL HIT applications. The specific aim of this study was to explore factors that providers consider when assessing the value added of an HRQL application for their geriatric patients.

**Methods:**

Three case studies were developed using the following data sources: baseline surveys with providers and staff, observations of staff and patients, audio recordings of patient-provider interactions, and semi-structured interviews with providers and staff.

**Results:**

The primary factors providers considered when assessing value added were whether the HRQL information from the module was (1) duplicative of information gathered via other means during the encounter; (2) specific enough to be useful and/or acted upon, and; (3) useful for enough patients to warrant time spent reviewing it for all geriatric patients. Secondary considerations included level of integration of the HRQL and EHR, impact on nursing workflow, and patient reluctance to provide HRQL information.

**Conclusions:**

Health-related quality of life modules within electronic health record systems offer the potential benefit of improving patient centeredness and quality of care. However, the modules must provide benefits that are substantial and prominent in order for physicians to decide that they are worthwhile and sustainable. Implications of this study for future research include the identification of perceived "costs" as well as a foundation for operationalizing the concept of "usefulness" in the context of such modules. Finally, developers of these modules may need to make their products customizable for practices to account for variation in EHR capabilities and practice workflows.

## Background

Incorporating new health information technology (HIT) into medical practice involves workflow changes and potential impacts, such as increased physician workload and compromised communication patterns between providers and patients. Although emerging HIT applications are directed to improving the quality of care and economies of service [1], some investigators have warned that the use of HIT may threaten the very nature of the patient-provider relationship by undermining trust, inhibiting disclosure of relevant concerns, and by hampering meaningful discussions of patient preferences that impact treatment decisions [2-5]. Reassuringly, HIT applications that incorporate point-of-care use of health-related quality of life (HRQL) assessments have been found to promote patient-centered interactions between seriously ill patients and physicians in specialty outpatient settings [6-9]. Although prior work has demonstrated the effectiveness of HRQL assessments in specialty clinics it is unclear what factors may contribute to the successful implementation of HRQL-HIT applications in primary care settings. For example, one prior feasibility study examining the use of an HRQL-HIT application in 14 German general practices found substantial variation between practice sites and identified time constraints as a substantial barrier to both collecting and effectively using the HRQL information [10]. Clearly, technological feasibility alone does not result in consistent use within real world practices.

To further explore factors affecting HRQL information use, we conducted a pilot implementation study [11] of an HRQL-HIT application in three primary care practices. We were particularly interested in understanding how physicians assessed the value of an HRQL application for their chronically ill geriatric patients. For these providers, geriatric patients are only a subset of their patient population; therefore, the HRQL application had to be embedded within the practices' existing electronic health record (EHR) systems in a way that it became available only for relevant patients.

To evaluate the implementation of the HRQL module, we used a comparative case study design. Our initial thinking about the implementation process was informed by the perceived attributes of innovations identified by Rogers [12]. However, our data collection was guided more specifically by the Technology Acceptance Model (TAM), which posits that a user's perceptions about the ease of use and usefulness of a technology influence the intent to use the technology, and ultimately actual use of the technology [13]. The specific aim of the study was to explore factors that providers consider when assessing the value added of a new HRQL information technology application. Our overall goal was to better understand how the practice environment and preferences of individual providers impact decisions about whether to use an HRQL EHR module.

## Methods

### Description of HIT Intervention

The Geriatric Enhancement Module (GEM) was developed by a team of university-based health services researchers in collaboration with a private software vendor. It is comprised of a 7-item questionnaire that gathers patient-reported health-related quality of life (HRQL) data about physical health, emotional health, physical functioning and limitations in activities of daily living, and level of social support. The goal of the GEM is improve the quality of care discussions among staff, providers and patients. The care settings in this study were the first to use the GEM.

The GEM software was designed so that items would be prompted to appear within the EHR during the intake portion of the medical encounter (i.e., when vital signs and chief complaints are recorded by clinical staff) for all patients 50 years and older. Patients viewed and entered answers to the GEM questions directly into the medical record system with the help of a research assistant (RA) and/or clinical staff person. During the patient encounter, GEM items were displayed in the EHR for the provider. Patients also could raise issues related to their answers to the GEM questions.

### Study design

In evaluating the value of using GEM, we were particularly interested in: (1) the practices' level of engagement in the GEM implementation; (2) the users' (i.e., providers and staff) opinions about their EHR system and expectations for the GEM; (3) the level of use of the GEM information during the patient encounter, and; (4) factors affecting the level of use and decisions about continuing to use the software. To structure our data collection and analysis, we used a multiple, embedded case study design with the embedded units being providers, nursing/administrative staff, and patients nested within the larger case (i.e., the physician practice). This design enables within and between case analyses [14]. Data collection for the study was staggered, beginning with one practice and expanding to others. This approach allowed for data collection procedures and/or sampling strategies to be modified as needed to account for issues that emerged [15].

### Selection and recruitment of study sites and patients

The study involved three primary care practices. Two were small (i.e., 1 or 2 providers), independently owned family practices located in small towns. The other was a general internal medicine practice that was housed within a large academic medical center. Given the focus of the study - HRQL modules within EHR systems - we needed to utilize practices that had operational EHRs that could incorporate the GEM. The two independently owned practices were recruited with the help of a medical software vendor that had a prior proprietary relationship with the practices and was knowledgeable about their IT capabilities. Based on initial findings from these first two sites, we sought variation in terms of ownership and size for the third site to explore the impact of these practice characteristics on the GEM implementation and assessment. Therefore, we recruited the practice from an academic health center with no current relationship with the vendor.

For all practices, the PI and other members of the project team visited the site to provide details about the study and obtained informed consent from members of the practice. After informed consent was obtained, a co-investigator and/or an (RA) with office nursing experience provided training to physicians and practice staff about the GEM. In addition to this training session, the RA was available for physicians and staff on designated data collection days to provide support and answer questions. Participating sites received a one-time $2500 incentive to reimburse them for staff time related to the study.

Clinical staff in the practices, with support from the RA, recruited a sample of 60 patient subjects for the study, approximately 20 from each practice site. No re-enrollment was permitted. Patients who met the following criteria were eligible for the study: (1) age 50 years of age or older; (2) self-reported diagnosis of heart disease, lung disease, stroke, or cancer, and; (3) capable of speaking and reading English language. Specific exclusion criteria for the study included: (1) severe memory loss or impaired orientation, and; (2) acutely ill appearing. Participating patients received a $10 gift card. The study was approved by the Institutional Review Board of the University of North Carolina at Chapel Hill prior to its initiation.

### Data collection and analyses

We used multiple sources of data to explore: (1) the practices' level of engagement in the GEM implementation; (2) the users' (i.e., providers and staff) opinions about their EHR system and expectations for the GEM; (3) the level of use of the GEM information during the patient encounter, and; (4) factors affecting the level of use and decisions about continuing to use the software. Our guiding framework for data collection about EHR opinions and GEM expectations, level of GEM use, and factors affecting GEM use was the Technology Acceptance Model (TAM) [13,16]. According to the TAM, two factors influence users' acceptance of new technologies: perceived usefulness and perceived ease of use. Perceived usefulness refers to the extent that a person believes that a particular system would enhance his or her job performance. Perceived ease of use refers to the extent that a person believes that using a particular system would be free of effort [13,16] (See Table 1.).

**Table 1:** Key Concepts and Data Sources

	Practice's level of engagement in GEM implementation	Provider/Staff EHR opinions and GEM expectations	Providers' level of GEM use	Factors affecting providers' GEM use
**Baseline Survey**	X	X		

**Direct Observation of Patient and Staff GEM Use**	X			

**RIAS Coding of Provider-Patient Interaction**			X	

**Semi-structured Interviews with Providers and Staff**				X

Prior to launching GEM in each practice, we administered a brief questionnaire to the physicians and staff who were likely users of GEM, or whose work potentially would be affected by use of the GEM in the practice. This questionnaire consisted of five-point Likert-type scales to gauge: (1) the perceived values related to HIT and patient care within the practice; (2) the perceived ease of use and usefulness of the practice's existing EHR system; (3) the awareness of the GEM intervention, and; (4) the expected ease of use and usefulness of the GEM. Patient recruitment began after the GEM was installed into each practice's EHR system. After informed consent was obtained, patients responded to the GEM items during the routine intake collection and recording of vital signs and chief complaint into the electronic health record. The RA was available to assist the clinical support staff and/or patient in GEM administration. After the intake collection was completed, the subsequent patient-provider encounter was recorded using a digital audio recorder. Immediately after the visit, the RA collected the audiotape and administered a post-encounter survey to patients, which asked about their use and satisfaction with GEM. We debriefed the RA about observations of the patients' and clinical support staff's engagement with the GEM.

Approximately three months after GEM patient data collection was completed, we conducted individual semi-structured interviews with providers and clinical/administrative staff from each site. A total of 16 interviews were conducted. The interviews explored the facilitators and barriers that users experienced in implementing GEM; their perceptions about the ease of use and usefulness of the GEM; the perceptions about the degree of alignment between the GEM and the users' values, and; recommendations for improving the GEM and promoting its sustainability. The semi-structured interviews were audio-recorded and professionally transcribed. In addition to these interviews, study staff recorded field notes about their general observations of the practice setting, as well as the practices' use of the EHR system.

Our approach to analyzing GEM's value-added included examining the level of engagement in the implementation, reviewing stakeholder opinions of the existing EHR and expectations for the GEM, measuring the level of use of the GEM, and identifying factors that influenced perceptions and use of the GEM. We assessed engagement in the implementation process both quantitatively and qualitatively via baseline surveys and direct observations. To measure provider usage quantitatively, we coded the patient-provider audiotapes using the Roter Interaction Analysis System (RIAS), a widely recognized method of coding doctor-patient interactions during the medical visit [17]. This analysis allowed us to gauge the level of GEM use at both the provider and practice levels. Specifically, RIAS measured provider references during the encounter to either GEM prompts in the EHR or to the GEM questionnaire administration and patient references to HRQL-related topics and the GEM administration. For provider measures, encounters were categorized by the number of references: 0 = no references, 1 = one reference, and 2 = two or more references. To gain a richer understanding of factors contributing to this variation, we analyzed the interview data, considering also the level of engagement and GEM expectations of each practice.

To assess each practice's satisfaction with their current EHR and expectations regarding GEM implementation, we analyzed data from the baseline surveys of providers, clinical staff, and administrative staff. These baseline questionnaires consisted of five-point Likert-type scales informed primarily by the TAM concepts of usefulness and ease of use. To identify factors affecting post-implementation perceptions and use of the GEM, we analyzed semi-structured interview data from providers and clinical/administrative staff collected approximately 3 months after GEM rollout. An investigator with expertise in health care innovation adoption and implementation (CS) initially coded the qualitative data from the interviews using the TAM framework [13,16] as a guide for developing the codebook. The analytic process involved memoing and identifying emergent codes [18]. It became clear during the case studies, that the providers were sole decision-makers about whether or not to continue using the GEM after the study period. We therefore focused our attention to the providers assessments of value added, which were based on perceived usefulness. To identify themes that provided insight into the factors considered by providers when assessing the value of the GEM and to help ensure internal validity [19], the entire research team reviewed the texts selected to illustrate the themes. The team, however, did not directly assess coding reliability for the entire interview transcripts.

## Results and discussion

### Description of the Sites Recruited

As discussed above, the first two practice sites were purposively selected based on similarities in size, ownership status, and previous relationships with the health IT vendor (Table 2).

**Table 2:** Implementation Site Characteristics

**Characteristic**	**Practice A**	**Practice B**	**Practice C**
Practice location and type	Suburb, private geriatric practice	Rural, private family practice	Small/medium city, academic internal medicine practice

Number of medical providers	2	1	102 (full & part-time)

Number of clinical support staff	5	3	13

Number of administrative staff	3	4	14

Annual patient visits	10,000	12,000	40,000

Payor mix (% of total patients)			
Medicare	23	25	34
Medicaid	2	15	8
Commercial insurance	70	40	37
Self-pay/uninsured	0	30	21

### Level of implementation engagement within practices

#### Practice A

The two providers in Practice A were very aware of plans to implement the GEM prior to the launch. Also, of the seven staff who participated in the study, three (including the practice manager) were very aware of plans for the GEM and four were not at all aware. In this site, the project team's RA trained the practice staff to use the GEM and provided consultation as needed. The staff were able to incorporate the GEM into their workflow without much difficulty.

#### Practice B

The single physician provider in Practice B was well aware of plans to implement the GEM. In addition, one of the four participating staff members was fully aware of such plans, with the others being somewhat or not at all aware. The project team's RA was directly involved in entering patient data for the GEM, even after completion of training sessions with staff. Several clinical staff acknowledged limited involvement with the GEM and heavy reliance on the RA.

#### Practice C

Only 1 of the 2 providers in Practice C who participated in the study was aware of plans to implement the GEM. In addition, the clinic administrator was the only non-provider study participant aware of GEM plans or involved in implementation. The decision to limit involvement with the GEM planning and implementation was made within the practice to avoid impeding workflow for the nursing staff and causing dissatisfaction. As a consequence, the RA was exclusively involved with all data collection in this practice site, and nursing staff assessments of ease of use and usefulness of the GEM were not available for Practice C. The rationale behind this decision was that the practice is involved with many research projects at any given time, which can create a burden for staff. Also, staff are employees of a large health care system with policies and protections not found in small independently owned practices. This organizational setting fostered a different environment for the implementation than that found in the other two study practices, specifically with respect to physician-nurse dynamics. However, it is notable that the nurse manager participated in a post-implementation interview about the purpose of the GEM and indicated that it fit well with the approach to care in the clinic.

### Provider and staff opinions about their EHRs and expectations of the GEM

There was substantial variation of opinion about their EHR system and expectations of the ease of use and usefulness of the GEM (Table 3).

**Table 3:** Provider Attitudes Regarding Electronic Health Records and Expectations of GEM

	Practice A: Provider 1	Practice A: Provider 2	Practice B: Provider	Practice C: Provider 1	Practice C: Provider 2
**Ease of HER**	Hard	Very easy	Somewhat easy	Somewhat Easy	Easy

**EHR Enables Tasks to be Completed**	Not at all	Somewhat	A great deal	A little	A great deal

**EHR Allows More Patients per Day**	Not at all	A little	A great deal	A little	A little

**EHR Enhances Effectiveness**	Not at all	Somewhat	A great deal	Somewhat	A great deal

**EHR is Patient Centered**	A little	Somewhat	A great deal	A little	A great deal

**Ease of Using GEM Info**	Very easy	Easy	Somewhat easy	Somewhat easy	Hard

**GEM Enables More Task Efficiency**	A little	Somewhat	A great deal	A little	A little

**GEM Enhances Effectiveness**	A little	A great deal	Somewhat	A little	A little

**Ease of Adjusting to the GEM**	Very easy	Very easy	Easy	Somewhat easy	Somewhat easy

#### Practice A

The providers had different opinions about how easy it was to operate their EHR system, whether the EHR enables accomplishing tasks, and whether the EHR enhances effectiveness. Both agreed that the EHR did not enable seeing more patients per day and generally agreed that the EHR collected patient-centered information but could be improved in that area. Also, both agreed that adjusting workflow to incorporate the GEM would be very easy, and expressed little or no concern about using the GEM information. However, one provider had higher expectations for the GEM to enhance their effectiveness.

#### Practice B

The provider clearly viewed the EHR system as a tool for improving efficiency, indicating that it enables accomplishing of patient care tasks and seeing more patients per day. He/she also viewed the EHR as a tool for enhancing effectiveness and considered it to be very capable of collecting patient centered information. In addition, the provider believed adjusting workflow to include the HRQL information would be easy, although it would be more difficult to use the information during the encounter. Finally, he/she expected the GEM to further enhance efficiency of tasks and effectiveness.

#### Practice C

The two providers reported that their EHR was "easy" and "somewhat easy" to operate, respectively. There was some disagreement about how well the EHR captures patient-centered information and how well the EHR accomplished patient care tasks but there was more agreement about the EHR enhancing effectiveness. Both acknowledged that the EHR did not substantially enable seeing more patients per day. They generally agreed that adjusting to having the GEM and using the HRQL information would not be particularly easy. Neither had high expectations for the GEM enabling them to accomplish tasks more efficiently or enhancing their effectiveness.

### Level of use of the GEM across practices and providers

Regarding providers' usage of the GEM information in discussions with their patients, there was substantial variation across providers even within the same practice. For example, Provider 1 in Practice A referred to the GEM questionnaire items during the patient encounter more than any other provider in our study, while Provider 2 in the Practice A never referred to the specific questionnaire items, although he/she did refer to the administration of the GEM questionnaire. Furthermore, Provider 1 in Practice C referred to the items and questionnaire administration more than Provider 2 in Practice C, who indicated in the baseline survey little awareness of plans to implement the GEM. Finally, the Provider in Practice B never referred to either the GEM items or the administration of the questionnaire in his/her discussions with patients, even though clinical staff and the RA incorporated the study patients' responses to the GEM items into the EHR (Table 4).

**Table 4:** Level of GEM Usage

		Provider						
		
		A	B	C	D	E	Total	P
Provider refers to GEM computer prompts *	Mean	0.75	0.00	0.00	0.50	0.08	0.28	< .001
	SD	0.68	0.00	0.00	0.84	0.29	0.56	

Provider refers to GEM questionnaire administration or computer prompts *	Mean	1.06	0.20	0.00	0.50	0.08	0.39	< .001
	SD	0.85	0.45	0.00	0.84	0.29	0.70	

Patient refers to GEM topics **	Mean	1.00	0.60	0.33	0.83	0.50	0.63	.15
	SD	1.03	0.55	0.69	0.41	0.67	0.79	

Provider/Patient refers to GEM ***	Mean	1.19	0.20	0.00	0.83	0.08	0.46	< .001
	SD	1.05	0.45	0.00	1.60	0.29	0.91	

* 0 = no, 1 = once, 2 = two or more times

** Count of times patient refers to one of the GEM topics (e.g., ADLs, quality of life)

*** Sum of times that either the provider or patient refers to the GEM computer prompts or questionnaire administration

### Factors affecting level of use and decisions to sustain use

None of the three practices continued to use the GEM after the study period. For each practice there were primary and secondary factors, which led to non-continuation of GEM use.

#### Primary Factor: The perceived benefits of GEM were not sufficient to warrant continued use

Judgments about perceived GEM benefits were influenced by the context of each practice, primarily the capabilities of the existing EHR and the providers' preferred approaches to patient encounters. These contextual differences influenced decisions about whether the GEM information was: (1) duplicative of information gathered via other means during the encounter; (2) specific enough to be useful and/or acted upon, and; (3) useful for enough patients to warrant time spent reviewing it for all geriatric patients.

##### Practice A

Practice A was technologically advanced and an early adopter of electronic health records. After implementation, the primary concern about the GEM was duplication of information collected by other systems and/or processes. For example, the practice's EHR had a module, in addition to GEM, that focused on geriatric patients. The GEM yielded some new information about a few of these patients; however, the providers perceived that it either did not yield enough valuable new information or it did not yield new information for enough patients to warrant continued use at this time.

"I felt like in most of the cases the issues that [GEM] discovered we already knew about or it came up in the course of the evaluation. There were a couple, and I think I actually pointed them out to the nurse when it did happen, where it was something surprising to me about the GEM Module. It did pick up something that we weren't aware of. And I think if we were going to use it, we would try to incorporate it more into our natural flow... Right here (pointing to the computer) everybody gets these four questions right now. It sort of gets some of the issues. And we also have for the geriatric patient; we have these questions that we ask them. If they've been in the hospital or if it's been a while, we do this once or twice a year. We go through this geriatric thing, which gets it a little bit more detailed than our usual interim history. And of course the GEM goes in a little farther than this" (Practice A, Provider 1).

The other provider expressed similar views:

"Maybe we discussed things a little more about socially and how certain things impact people, but I think that we have a tendency to try to really focus on that. For instance, we will use our [current EHR capabilities] with diabetes to try and find out how it would impact you psychologically. And for some people we have actually found that it really stresses them out about their diabetes, whereas other people it doesn't. So we've been trying to do some of that sort of thing" (Practice A, Provider 2).

While providers recognized some value in the GEM information, they were not compelled to continue using it, primarily because the practice already had processes in place for collecting similar information for its geriatric patients.

##### Practice B

Practice B used its EHR system as an efficiency tool to manage a high volume ofpatients. Despite pre-implementation optimism about the ease of using GEM information, the provider in this practice never mentioned the information during discussions with patients. The provider's primary concern about the GEM after implementation was that it did not elicit specific types of information to enhance what was already being collected by the practice's systems and processes:

"It's watered down compared to what I was using... I wasn't getting the wealth of information... So, I did not find using a sole module of value as opposed to utilizing an entire system" (Practice B, Provider).

Since we did not directly observe the information accessed by the provider in the EHR during patient encounters, we do not know if he/she saw the GEM information and chose not to refer to it with patients, or whether he/she ever viewed the GEM information at all. Based on the provider interview and our understanding of the practice workflow, which involved nursing staff inputting, summarizing and/or highlighting information in the EHR for the provider, the provider may not have had a clear understanding of which pieces of information were resulting from the GEM.

##### Practice C

The providers in Practice C were not optimistic about the usefulness and ease of using the GEM information. Their skepticism was due to both organizational factors at the practice level and individual provider preferences. From an organizational perspective, the practice, based within an academic medical center environment, had many competing demands due to research projects and quality improvement initiatives. Along these lines, the primary concern expressed by the providers was that the GEM information's value did not clearly outweigh the time involved to review and use it. One provider focused on targeting GEM to the patients for whom it would yield useful information.

"I like the idea of being targeted to an older population and functional status because I think we often overlook that. I think the measure is good... I think my advice would be to target to a more likely population that's going to have some functional problems... because I think if you're giving physicians a bunch negative information, it's not particularly helpful... So if there's no functional impairment and I wouldn't expected there's functional impairment in that patient, then that's not really helping me right? It's just increasing my time and slowing me down" (Practice C, Provider 1).

The second provider focused on the fit with his/her preferred practice style and the opportunity cost of using the GEM:

"Let me tell you a little bit about my practice style and why I think it's hard to incorporate anything else into the practice. And one of the things is that we're tied to, for the most part, is the 20 minutes office visit, and one of my personal characteristics is not to wanting to get behind. So I try not to be more than 15 minutes behind during a clinic schedule. Sometimes I fail. So all that said I think the most important part of the time with patients is just an open-ended interview for a bit of time, and that is a priority for me, and I find a lot of additional things burdensome, helpful but burdensome" (Practice C, Provider 2).

However, the provider acknowledged seeing some value in using the GEM for improving patient care.

"I think for a subset of patients on that day it made me think more about how they're functioning at home and if they need more support... I think that was the major benefit that I saw. Now I think it is a benefit, the question is, the question that you and others will need to decide is, what are the relative benefits of doing this vs. doing other things" (Practice C, Provider 2).

The clinic administrator, who serves on the clinic's research committee, supported this perspective of competing research demands and the importance of physician perceived benefit in any new workflow process:

"Again the value that the provider sees is key. As I say their mindset is what more can we do? So if they saw this as a successful tool either to be used in whole or part my guess is that they would be interested... We have a very active QI process in the clinic and new things come up all the time" (Practice C, Clinic Administrator).

Interestingly, one of the providers indicated that a barrier to the GEM's sustainability was that it was developed by researchers not affiliated with the practice.

"I think it's really hard coming in from the outside to try to implement something. I'm not sure that we know how, we as in the real world know how to do that" (Practice C, Provider 1).

#### Secondary factors: system integration, nursing workflow, and patient reluctance

Three additional factors contributed to the practices' level of GEM usage and decision to sustain usage: (1) level of integration of the GEM information into the EHR system; (2) consistency of data collection process with nursing workflow; and (3) patient reluctance to provide HRQL information. (See Table 5 for illustrative quotations.)

**Table 5:** Factors Contributing to the Value-Added of GEM

Theme	Practice A	Practice B	Practice C
**Level of integration**	"The whole thing when we bought this particular program [EHR name], they told us well the next version [program name] will be fully integrated. That's I don't know how many versions ago, and it's not, and it never will be as far as I can tell... I told the [researchers from another study] if you just get [the vendor] to write a little subroutine to pull this stuff out so it was actually in the system, I think we would use it every time we did a lipid panel on somebody. But trying to get the stuff you guys do into these commercial vendors software is difficult." (Provider 1)	"Well, we've used the [EHR modules] with the patient-entered questionnaire since 2003. And so the GEM was more like a modification of the same program... Technically, the only challenges that we had was in 2005 the computer vendor could not integrate all the questions that came out of [the EHR modules] into the computer vendor's software as part of the HPI, so what we had to do was literally cut and paste in blocks those responses out of [the EHR modules] into the HPI section coming in as one data point... It is still not integrated" (Provider).	

**Nursing workflow**	"It was similar to what we do already for the questions we ask... I didn't find it hard to use" (Staff 2).	"Say if I have a diabetic patient and I have to do a lot with a diabetic patient that hasn't been in the office in a while and if I'm doing a lot, like, they might need an EKG for the exam here and blood sugar you know, just a lot, then they bring like a bunch of medicine and you have to key in all their medicine. It's just time-consuming... you know, you're trying to work as fast as you can because you've got other patients in the lobby ready to come back" (Staff 4).	"If I had to ask the [GEM] questions I would not be happy about that" (Provider 2). "I can see if a nurse, or a care provider, or somebody ahead of time was... asking [the GEM] questions, that that could be an issue... They just don't want to have to sit in there for 30 minutes, you know, because they're trying to get people checked in and out" (Provider 2).

**Patient Reluctance**	"I think we probably lost some patients when we first implemented [our EHR]... Oh yeah, absolutely... No, they're all gone. Those people that (pause) I had a friend of mine that said if he had a doctor that typed while he was being seen that he would just go to another doctor. He just thought it was totally inappropriate. I can't argue with that. But this particular practice after five years of this stuff is gonna object to what, 8 questions, or whatever it is? No" (Provider 1).	"We also ran into issues, which was surprising, of patients saying, 'Well I don't want to put my information in the computer.' Well you put them in IMH last week! But this week you don't want to do the GEM module because somebody came in and said, 'I'll give you a ten-dollar Wal-Mart card if you're part of the study.' Whereas last week it was IMH that was part of the routine of the practice. And so, it was something about being a research person or whatever that (pause). Again, these were all study issues that came up, but not, not the module" (Provider).	

##### Level of integration of the HRQL information into the EHR system

Practices A and B were concerned about how well components of the current EHR were integrated, and how well the GEM was integrated into the EHR so that information could be accessed easily. This was not a concern in Practice C, which was notable because that practice used a different method for integrating the GEM into the EHR, specifically a text editing software program.

##### Consistency of clinical data collection with nursing workflow

GEM fit well with the nursing and office staff workflow in Practice A. In Practice B and Practice C, there were concerns about the GEM increasing nursing workload. Possible explanations for the varied perceptions include differences in organizational culture, workflow efficiency, and nursing staff experience and capabilities.

##### Patient reluctance to provide HRQL information

In Practice A and Practice C, patient reluctance to answer the GEM questions was not viewed as an issue by providers and staff. However, patient reluctance, particularly among low literacy patients, was a common topic of discussion in interviews with the Practice B provider and staff. We were uncertain as to the factors that contributed to this perception. Some possibilities include differences in patient populations (e.g., in literacy levels and skepticism about medical research) and how the opportunity to participate in the GEM study was presented.

## Conclusion

Implementing a new EHR module that focuses on patient-entered HRQL information within a primary care practice has implications for providers, staff, and patients. From our data, providers' perceptions about the module appear to have the strongest influence on decisions about whether the module should be used in the practice. In addition, our exploratory findings study shed light on the factors providers consider when determining the value-added of an HRQL EHR module.

One framework for understanding the value of a product or service, such as HIT, is to calculate its "quality" divided by "cost" (Value = Quality/Cost) [20]. Implicitly, the practices' stakeholders (i.e., providers and staff) in this study made a similar assessment about the value added by the GEM. Their primary concern (i.e., the relative advantage of using the GEM vs. the status quo EHR) relates to the GEM's impact on the quality of the service provided during the encounter. For providers, criteria for assessing this impact were the extent to which the GEM information was duplicative of information gathered elsewhere, specific enough to be acted upon, and applicable for enough patients. This was a different assumption by our research study staff since we hypothesized that a perceived benefit of the GEM would be supportive documentation for enhanced billing and coding. However, none of the practices saw this as a benefit, either because there was not a perceived need for it or the GEM was not sufficiently integrated into the billing system. The opportunity for the GEM clearly was in the realm of improving the quality of patient care.

The secondary factors that emerged (i.e., EHR integration, nursing workflow, and patient reluctance) relate to the cost of using the GEM once implemented. For integration, the cost is lost time during an encounter due to inadequate integration of the module into the EHR (or the cost of achieving better integration, if possible). Successful integration of the module is made even more difficult when providers do not perceive the original functions of the EHR system (e.g., history of present illness, medications list, etc.) to be well integrated. This lack of integration leads to fragmentation of the EHR and, subsequently, to "cutting and pasting" of information and/or toggling between screens. In such cases, adding the HRQL module runs the risk of causing further EHR system fragmentation.

For staff and patients, such a module can affect workflow and communication patterns during the visit. If nursing staff must spend more time asking the HRQL questions or assisting patients with data entry of their responses, there could be a negative impact on patient throughput and, ultimately, on staff job stress and satisfaction. Furthermore, there is some evidence that staff satisfaction is positively correlated with patient satisfaction [21]. Clearly, a perception that the GEM could result in increased staff turnover costs or lost business for the practice would be a significant barrier to implementation. In summary, the cost concerns illustrate the need for any HRQL module to align with the current EHR system design, nursing staff capabilities and workflows, and patient preferences.

The findings from this study inform future research on EHR implementation in a several ways. First, the Technology Acceptance Model concept "usefulness" is multi-dimensional and context dependent. For example, a technology may be useful because it increases an individual's effectiveness or efficiency, or both, depending on the purpose of the technology, the user's role, and the setting [22]. The criteria providers used to determine the impact of the GEM on the quality of care delivered (i.e., information duplication, specificity, and applicability to enough patients) could assist researchers with operationalizing "usefulness" in the context of HRQL EHR modules. Second, the providers' cost concerns illuminate a range of costs that must be mitigated in order for an HRQL module to be accepted. These findings are consistent with those of Rogausch and colleagues [10], as the costs all relate to time constraints. Clearly, the HRQL module must be well integrated into both the EHR and into the workflow. From a research perspective, these are distinct but related concerns and both should be measured. Third, this study illustrates that EHR module implementation is influenced by both organizational (i.e., practice-level) and individual (i.e., provider-level) factors [23]. Even practices that are similar across important characteristics (e.g., size and years experience with EHRs) will have substantial variation in priorities and workflows. Providers within the same practice may have different views about their EHR system and the need for an HRQL module. Future research should include both the practice and provider levels of analysis. Furthermore, HRQL interventions may need to be customizable to meet the needs of different practices and providers.

This study had a few limitations. First, the project team was responsible for developing the GEM module, recruiting participating practices, assisting with implementation, and assessing the module (e.g., uptake and provider satisfaction with it). This involvement potentially could have resulted in a positive response bias, but this does not appear to have occurred in this case. However, assisting with implementation and data entry into the GEM may have resulted in an easier implementation process for the study participants than for practices that might implement the GEM without such support. Second, not having the nursing staff in Practice C available to participate in the study was a data limitation for assessing the impact of the GEM on workflow in that practice. However, the rationale provided by the practice for not including the nursing staff was useful data in itself for assessing the practice's culture and level of engagement in the GEM implementation. Third, while we were able to code audio data of provider-patient communication to assess the level of GEM usage, we were not able to observe how the provider accessed the GEM in the EHR. Differences in how providers incorporated the GEM into their workflow may have affected their assessment of its ease of use and the value of its information. Fourth, the providers were not provided with information about patients' perceptions of the value of the HRQL module, which generally were positive. Providers' considerations of value added might have been different if they were aware of these patient perceptions. Finally, since the study evaluated the feasibility of implementing the GEM in only three practice sites, the findings may not reflect all important factors considered when assessing the value added of an HRQL EHR module in primary care practices. However, the study provides a foundation for larger studies aiming for generalizability. Our study also identifies issues to consider for developers of HRQL modules [24].

In summary, our findings suggest that any EHR enhancement must have perceived value that justifies the investment. Plausibly, a benefit of an HRQL module is improved quality of patient care; however, in busy practice settings, we found that providers and staff are skeptical of adding another activity to complete during the patient visit. In addition, some providers may not believe that including structured HRQL information in the EHR is the most effective method for improving patient centeredness. Therefore, the benefit of new EHR modules must not only be present, it must be prominent for providers and align with their priorities and workflows. For some practices, this prominence perhaps could be achieved by framing the HRQL module as a method for obtaining external incentives (e.g., Meaningful Use of EHRs). However, achieving alignment with priorities and workflows is still a complex, context-dependent process.

## Abbreviations

EHR: Electronic health record; GEM: Geriatric Enhancement Module; HIT: Health information technology; HRQL: Health-related quality of life

## Competing interests

The authors declare that they have no competing interests.

## Authors' contributions

CS participated in designing the study, led analysis of qualitative interview data, and drafted the manuscript. JH participated in designing the study, debriefed the research assistant to capture observational data, validated themes identified in the qualitative analysis, and assisted with editing the manuscript. DR provided analysis of quantitative data and assisted with editing the manuscript. TD participated in designing the study, validated themes identified in the qualitative analysis, and assisted with editing the manuscript. All authors read and approved the final manuscript.

## Appendix A. Appendix

The GEM is comprised of a 7-item questionnaire that gathers patient-reported quality-of-life data about physical health, emotional health, physical functioning and limitations in activities of daily living, and level of social support. The goal of the GEM is improve the quality of care discussions among staff, providers and patients.

### The GEM Module Questionnaire

In the last 4 weeks, how much have emotional problems, such as feeling anxious and irritable, or feeling downhearted and blue, been bothering you?

Not at all

A little bit

A moderate amount

Very much so

All the time

In the last 4 weeks, how much difficulty have you had doing your usual activities, such as family activities, work, or housework, due to your physical or emotional health?

No difficulty at all

A little bit of difficulty

A moderate amount of difficulty

A great deal of difficulty

I cannot do these activities

In the last 4 weeks, how much difficulty have you had caring for yourself, such as eating, bathing or showering, dressing, and getting around?

No difficulty at all

A little bit of difficulty

A moderate amount of difficulty

A great deal of difficulty

I cannot do these activities

Do you currently have someone available who would take care of you if you wanted and needed help?

Yes, for as long as I needed

Yes, for a long time, such as 3 to 6 months

Yes, for a moderate time, such as less than 3 months

Yes, for a short time, such as less than a month

No, there is no one available

In the last 4 weeks, how much has your physical and emotional health gotten in the way of your ability to get together with family and friends?

Not at all

A little bit

A moderate amount

Very much so

All the time

In the last 4 weeks, how would you rate your overall health?

Excellent

Very good

Good

Fair

Poor

In the last 4 weeks, how much have health care concerns, such as going to the doctor or hospital, been on your mind?

Not at all

A little bit

A moderate amount

Very much so

All the time

## Pre-publication history

The pre-publication history for this paper can be accessed here:

http://www.biomedcentral.com/1472-6963/12/67/prepub
